# Removal of subretinal proliferative vitreoretinopathy: case series and literature review

**DOI:** 10.1186/s40942-025-00704-2

**Published:** 2025-07-15

**Authors:** Sukhum Silpa-archa, Pawas Lalitwongsa

**Affiliations:** 1 Department of Ophthalmology, Rajavithi Hospital, College of Medicine, Rangsit University, 2, Phayathai Road, Ratchathewi District, Bangkok, 10400 Thailand; 2 Department of Ophthalmology, Priest Hospital, 445, Si Ayutthaya Road, Ratchathewi District, Bangkok, 10400 Thailand

**Keywords:** Proliferative vitreoretinopathy, Subretinal proliferation, Aspiration cutter, Vitrectomy, Retinal detachment

## Abstract

**Background:**

To present the results of a simplified technique for removing subretinal proliferation bands (SPB) using membrane forceps and a vitrectomy aspiration cutter.

**Methods:**

Retrospective interventional case series.

**Results:**

Out of 241 eyes (241 patients), 31 (13%) had SPB detected preoperatively or intraoperatively and underwent pars plana vitrectomy with SPB removal using membrane forceps and an aspiration cutter. Of these 31 eyes, the most common PVR grade was C3 (39%), while the most severe was D1 (10%). 61% of eyes underwent a combined scleral buckling procedure, and all eyes were tamponaded intraoperatively. SPB was completely removed in 74% (23/31) of cases. Intraoperative complications were detected in 10% (3/31): retinal hemorrhage (2/31) and subretinal hemorrhage (1/31). 84% (26/31) had complete retinal reattachment after a median follow-up time of 10 (range, 2–32) months, and 74% (23/31) of patients reported an improvement in BCVA after surgery.

**Conclusions:**

Conclusions: For SPB removal, the use of an aspiration cutter can improve grasping of the band and its stump, reduce band fracturing, and minimize instrument exchanges during removal.

**Supplementary Information:**

The online version contains supplementary material available at 10.1186/s40942-025-00704-2.

## Background

A subretinal proliferation band (SPB) is a type of proliferative vitreoretinopathy (PVR) that develops following a rhegmatogenous retinal detachment. It was first defined by Duke-Elder and Dobree in 1967 [1]. SPBs are categorized into two main types. The first type, predominantly composed of glial tissue without contractile force, tends to form diffuse cell sheets that do not hinder retinal reattachment, while the second type contains differentiated retinal pigment epithelium with myofibroblasts that exert contractile force on the retina, leading to taut membranes or bands. This second type often impedes retinal reattachment and typically requires removal [2, 3]. Lewis and coworkers pioneered the study of subretinal membranes in PVR and discovered that in 28% of 72 eyes, SPB impeded retinal reattachment and had to be removed or excised via a preexisting retinal break or one or more retinotomies [4]. There have been several surgical procedures documented for removing the SPB. These include the conventional method through the vitreous cavity [5–8] and the subretinal approach [9–13]. The transvitreal method involves inserting intraocular forceps into the subretinal space via preexisting retinal breaks or a small retinotomy site created by endodiathermy [4]. Furthermore, a lighted pick or two membrane forceps can be utilized for a hand-over-hand technique, and a bimanual method with chandelier illumination is also an option. The subretinal approach can be accomplished via subretinal peeling [9, 13] or endoscopic vitrectomy [11]; this requires an extra scleral puncture with a trocar positioned 9 mm to 16 mm from the limbus.

For transvitreal SPB removal, Hahn P. was the first to propose using a vitrectomy aspiration cutter in addition to membrane forceps [14]. The unimanual “Spaghetti Twirl” technique, which involves wrapping membranes around the vitreous cutter for SPB removal, has not previously been detailed in terms of its surgical steps or clinical outcomes. Our study offers the first formal, step-by-step description of this transvitreal approach, integrating the use of membrane forceps and an aspiration cutter as applied in our cases. This technique constitutes a simplified method of SPB removal. Additionally, we present the treatment outcomes of 31 patients who underwent SPB removal using this approach and include a comprehensive review of the literature on surgical techniques for this procedure.

## Methods

This study was conducted at the Ophthalmology Department, Rajavithi Hospital, Bangkok, Thailand. It followed the tenets of the Declaration of Helsinki and was approved by the Ethics Committee of Rajavithi Hospital (no. 227/2567). Patients giving informed consent for participation were enrolled from October 2021 to August 2024.

The search included publications on the PubMed database from 1968 to February 2025. Search terms included “proliferative vitreoretinopathy”, “subretinal proliferation bands”, “subretinal proliferation tissue”, “subretinal bands”, “subretinal strands”, “subretinal membranes”, “subretinal proliferation”, “subretinal surgery”, “proliferative diabetic retinopathy”, and “retinal detachment”. Inclusion criteria were any relevant articles in English published up to February 2025.

### Patient characteristics

A retrospective chart review was conducted of cases with rhegmatogenous retinal detachment undergoing vitrectomy performed by an experienced vitreoretinal specialist (S.S.) at the Ophthalmology Department, Rajavithi Hospital, between October 2021 and August 2024. Among 241 eyes from 241 patients, SPB was identified preoperatively or intraoperatively in 13% (31/241) of cases through dilated fundus examinations performed by the same specialist (S.S.). All patients underwent pars plana vitrectomy with SPB removal using membrane forceps and an aspiration cutter. (Table 1) Each case had first surgery performed for rhegmatogenous retinal detachment, except for two eyes where the retina was not reattached after recent surgery. The grade of PVR was determined according to the 1983 classification of retinal detachment with proliferative vitreoretinopathy [15]. The most common PVR grade was C3 (39%, 12/31), while the most severe was D1 (10%, 3/31). The criteria for SPB removal were as follows: (1) pigmented annular or multiple branching configurations; or (2) significant retinal tautness persisting after conventional maneuvers. A majority of patients (52%, 16/31) experienced SPB encompassing two quadrants, and submacular SPB was detected in 10% (3/10) of patients. Prior to surgery, the median best-corrected visual acuity (BCVA) was 20/2000 (2.0 LogMAR).

**Table 1:** Demographic characteristics and treatment outcome of 31 cases undergoing subretinal proliferation band removal using membrane forceps and an aspiration cutter

	Cases undergoing SPB removal *N* = 31 (%)
Female	12 (39)
Age, year, median (range)	61 (20–86)
LogMAR BCVA at presentation, median (range)	2.00 (0.40–2.90)
SPB presented preoperatively	14 (45)
PVR grade	
C1	7 (22)
C2	9 (29)
C3	12 (39)
D1	3 (10)
Extent of SPB, quadrant, median (range)	2 (1–4)
Presence of submacular SPB	3 (10)
Operation	
Combined phacoemulsification	10 (32)
Combined scleral buckling procedure	19 (61)
Internal limiting membrane peeling	12 (39)
Tamponade with silicone oil	16 (52)
Complete removal of SPB	23 (74)
Intraoperative complications	
Retinal hemorrhage	2 (6)
Subretinal hemorrhage	1 (3)
Final follow-up	
Complete retinal reattachment	26 (84)
LogMAR BCVA, median (range)	1.00 (0.20–2.30)
Follow-up duration, months, median (range)	11 (2–38)

SPB = subretinal proliferation band, BCVA = best-corrected visual acuity, PVR = proliferative vitreoretinopathy

### Surgical technique

**Fig. 1:**
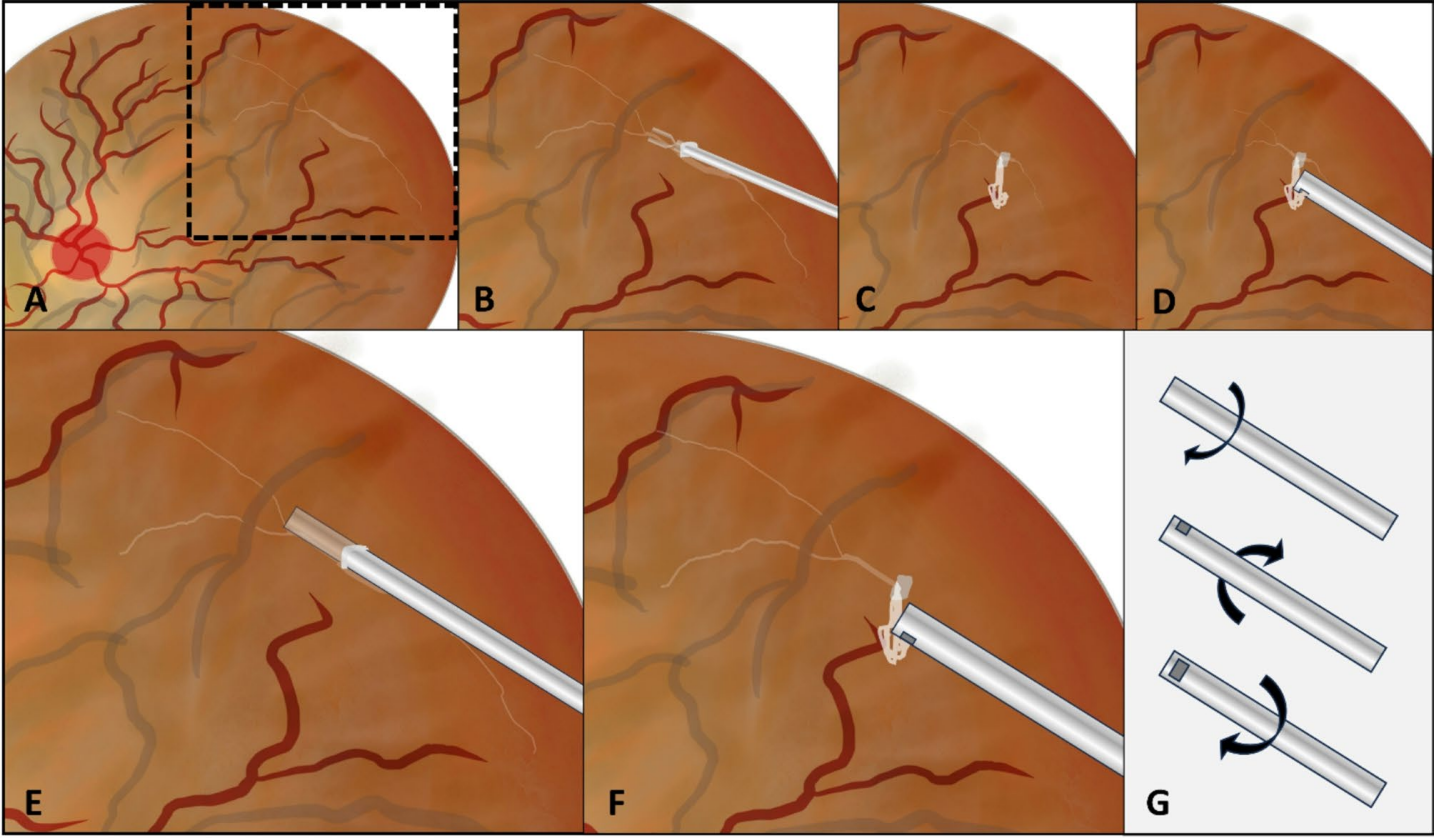
Schematic diagrams illustrate the technique. (**A**) A branching subretinal proliferative band was found underneath the detached retina (dotted box). (**B**) The forceps were inserted into the subretinal space through the retinal hole, grasping the expected point of the band. (**C**) Following the removal of the proliferative band from the subretinal space, a stump was left in the vitreous cavity, which should be at least 2–3 mm long to allow the cutter to aspirate without damaging the retina. (**D**) The stump was engaged by the cutter’s port using the cutter’s aspiration (cutter-off mode). At this stage, the cutter allows controlled engagement of the tissue, similar to manual grasping with forceps. (**E**) When the band was prematurely severed and retracted into the subretinal space, the cutter’s port was turned down and directed toward the free-ending stump. Vacuum aspiration was gradually increased until the subretinal proliferation stump became engaged. (**F**) The proliferation band was carefully pulled into the vitreous cavity using the cutter’s aspiration. (**G**) The “Spaghetti Twirl” technique involved continuously rotating the cutter’s tip while applying active aspiration, mimicking the motion of twirling spaghetti to remove the band

A standard 3-port 23-gauge pars plana vitrectomy was performed utilizing the Constellation Vitrectomy System (Alcon Laboratories, Inc, Fort Worth, TX) with a sutureless and equipment-free technique for contact lens wide-angle viewing system [16]. Initially, core vitrectomy was performed and epiretinal proliferation was removed. If the primary retinal break was not suitable for the removal of SPB, a retinotomy was created adjacent to or above the SPB. Penetrating diathermy was carried out with care taken not to sever the SPB below. A pair of membrane forceps was introduced into the subretinal space through the retinal hole, grasping the expected point of SPB, and then tangentially pulling it out into the vitreous cavity. (Fig. 1A and B) The short SPB was usually removed with a single grasp of the forceps. However, lengthy or branching SPB proved difficult to remove completely even when using the bimanual technique with chandelier illumination. Therefore, a vitrectomy cutter could be useful at this stage. 

The first attempt with forceps may not entirely remove the band, leaving a ripped stump of SPB anterior to the retinal break that can be easily caught by the aspiration cutter. (Fig. 1C) This cutter (cutter-off mode), employing a vacuum of at least 200 mmHg, is proportionally increased near the stump to engage the SPB; at this stage, the cutter allows controlled engagement of the tissue, similar to manual grasping with forceps. (Fig. 1D) The tissue is then removed by moving the cutter, rotating the port, raising the vacuum, or a combination of these actions, allowing the band to retract into the cutter’s lumen. This maneuver utilizes vacuum aspiration to provide a proportional pulling force without the compressive force generated by forceps when grasping the SPB, which can damage and tear the SPB. (see Video 1, which demonstrates utilization of an aspiration cutter for subretinal proliferative band removal in vitreous cavity) Lengthy or branching SPB was also simply managed using this technique because the detached band was removed without withdrawing instruments from the eye, potentially reducing surgical time and lowering the risk of intraoperative complications. Furthermore, if the band was prematurely severed and retracted into the subretinal space leaving the free-ending stump, it was difficult to handle with the forceps, and the aspiration cutter could be useful in this scenario. To regain the fragmented SPB, the cutter’s port was turned down and introduced into the subretinal space toward the free-ending stump. (Fig. 1E) (see Video 2, which demonstrates utilization of an aspiration cutter for subretinal proliferative band removal from subretinal space) The vacuum aspiration was then gradually increased until the SPB stump was engaged, and the cutter’s tip was brought through the retinal hole into the vitreous cavity. (Fig. 1F) The cutter’s aspiration force was sufficient to entirely remove the remaining SPB. If the cutter’s lumen becomes partially occluded, the “Spaghetti Twirl” approach may be useful. While the SPB was engaged, the approach involved continuously rotating the cutter’s tip with one hand while applying active aspiration, mimicking the motion of twirling spaghetti to remove the band. (Fig. 1G) If the cutter’s lumen becomes completely occluded, the cutter-on mode can be used to cut the band, and the newly-formed stump can be removed using the cutter’s aspiration. It is essential to ensure that the cutter’s port remains away from the detached retina during this maneuver.

## Results

Table 1 shows the treatment outcomes of 31 eyes that had SPB removal with membrane forceps and an aspiration cutter. In this cohort, 32% of eyes underwent combined phacoemulsification, while 61% underwent scleral buckling. All were tamponaded intraoperatively, with silicone oil used in 52% of cases and perfluoropropane gas in 48%. SPB was completely removed in 74% (23/31) of cases. Intraoperative complications were detected in 10% (3/31): retinal hemorrhage (2/31) and subretinal hemorrhage (1/31). The retinal hemorrhage was minimal, and it was stopped and removed intraoperatively. A case was reported in which an intraoperative subretinal hemorrhage was completely removed with the aspiration cutter during fluid-air exchange. Neither relaxing retinotomy nor retinectomy was performed in any of the cases. 84% of eyes (26/31) had complete retinal reattachment after a median follow-up time of 11 (range, 2–38) months. Retinal reattachment failure was observed in 16% (5/31) of cases, all of which were tamponaded with silicone oil; among these, silicone oil was retained in three cases. Of the 16 eyes tamponaded with silicone oil, silicone oil was removed in 69% (11/16) of cases, and the retina remained attached in 82% (9/11) of those cases. The mean and median final LogMAR BCVA of the patients were 1.28 ± 0.70 and 1.00, respectively, with a range of 0.20 to 2.30. The Wilcoxon Signed Ranks Test showed a substantial improvement in BCVA at the final follow-up compared to preoperative values (*P* < 0.01). 74% (23/31) of patients reported an improvement in BCVA after surgery.

## Discussion

Addressing the challenges of SPB management requires careful initial engagement and avoidance of premature severing of the band before its complete removal. Various techniques have been employed, including the use of intraocular forceps through a primary retinal break or a created retinotomy [4, 8], or the use of a subretinal approach [9–13] (Table 2). While the combination of a vitrectomy aspiration cutter with membrane forceps—such as the “Spaghetti Twirl” technique—has long been recognized for its simplicity and advantages, there has been no prior detailed documentation of its surgical steps or clinical outcomes [14]. This method offers several key benefits: (1) the cutter’s port geometry allows for efficient engagement, reducing the need for repeated gripping with forceps; (2) it avoids the compressive force of forceps that could damage or prematurely sever the SPB; and (3) it permits SPB removal without the need to withdraw instruments from the eye.

**Table 2:** A review of literature on the surgical technique of subretinal proliferation removal

	Transvitreal approach			Subretinal approach	
	Membrane forceps and cutter’s aspiration (Author’s)	Membrane forceps and lighted pic	Bimanual technique with chandelier illumination	Subretinal peeling	Endoscopic technique
Visibility through a typically detached retina	Optimal	Optimal	Optimal	Fair	Minimal
Learning curve	Shallow and short	Shallow and short	Steep and short	Steep and short	Steep and long
Advantages	-Semi-experienced surgical skill is needed -Requires only basic vitrectomy instruments -Reduced risk of losing or cutting membrane	Semi-experienced surgical skill is needed	-Semi-experienced surgical skill is needed -Reduced risk of expanding the retinotomy	-No need for retinotomy -Indicated for treatment of subretinal residual perfluorocarbon heavy liquid and silicone oil	Indicated in cases of severe corneal opacities or opaque retina
Limitations	-Accidental widening of the retinotomy -Secondary PVR and postoperative visual field loss owing to retinotomy -Increased risk of retinal injury, particularly during the removal of subretinal PVR using a cutter	-Requires a lighted pick -Accidental widening of the retinotomy -Secondary PVR and postoperative visual field reduction owing to retinotomy	-Requires a chandelier illumination and extra forceps -Accidental widening of the retinotomy -Secondary PVR and postoperative visual field reduction owing to retinotomy	-Upper-intermediate surgical skill is needed -Needs an additional trocar with cannula -Limited peeling space -Higher risk of complications (retinal and RPE damage, subretinal bleeding, and retinal incarceration) -Not appropriate for cases with severe PVR	-High cost -Advanced surgical skill is needed -Needs two additional trocars with cannulas

PVR = proliferative vitreoretinopathy, RPE = retinal pigment epithelium

Wolff was the first to describe a method for accessing the subretinal space without the need for a retinotomy [10]. This technique involved a transscleral vitrectomy for grade C PVR cases, allowing SPB removal via an extra scleral trocar. While this approach presents a potential alternative to the transvitreal method for subretinal fibrosis removal and fluid drainage [9, 12, 13], it is based on limited case series, involves a steep learning curve, and carries risks such as subretinal hemorrhage and retinal incarceration. Another option is the subretinal endoscopic technique, which uses an ophthalmic endoscope inserted beneath the detached retina to directly remove the SPB under endoscopic visualization without requiring retinotomies [11]. Although this approach has demonstrated safety and effectiveness in treating rhegmatogenous retinal detachment with grade C PVR, it demands expensive surgical equipment and advanced skills. In contrast, the combination of a vitrectomy aspiration cutter and membrane forceps offers a simpler alternative. This technique has a shallow learning curve, relies on standard vitrectomy instruments, and has been successfully adopted by many vitreoretinal fellows during their training.

The major disadvantages of this approach are the creation or expansion of the retinotomy in some cases, as well as an increased risk of retinal damage, particularly when removing subretinal PVR with a cutter. (Table 2) The favorable condition for the subretinal approach is a highly elevated detached retina, and thick or long SPB which completely occludes the cutter’s port. When the cutter’s tip is in the subretinal space, the port should be turned down and away from the detached retina, and the cutter-off mode should be confirmed before starting the vacuum. When pulling the cutter’s tip from the subretinal space back to the vitreous cavity, vacuum aspiration should be reduced, but not stopped, when passing through the retinal hole. In addition, maintaining a wide-angle view while removing the band, particularly for thick, lengthy, and branching SPB, is mandatory. For submacular SPB removal, as demonstrated in Video 2, the subretinal approach remains a viable option. However, in cases involving a thick and opaque retina, the endoscopic technique may be preferable to the transscleral approach for subretinal peeling due to its superior visibility.

In conclusion, we present the treatment outcomes of a simplified method for removing subretinal proliferation using membrane forceps and a vitrectomy cutter, along with a comprehensive review of surgical approaches for SPB removal. This technique improves the grasping of bands and their stumps, minimizes band fracturing, and reduces the need for instrument exchanges during the procedure. It is straightforward enough to be incorporated into fellowship training and is highly applicable in routine surgical practice, as it does not require specialized equipment, making it accessible to most vitreoretinal surgeons.

## Electronic supplementary material

Below is the link to the electronic supplementary material.

Supplementary Material 1: Video 1: Removing the subretinal proliferation band.

Supplementary Material 2: Video 2: Submacular proliferation membrane removal.

## Data Availability

No datasets were generated or analysed during the current study.

## Abbreviations

SPB: Subretinal proliferation band
PVR: Proliferative vitreoretinopathy

## References

[1] Duke-Elder S, Dobree JH. The formation of avascular connective-tissue bands in proliferative diabetic retinopathy. Bibl Ophthalmol. 1968;76:133–8. 5674833

[2] Hiscott P, Grierson I. Subretinal membranes of proliferative vitreoretinopathy. Br J Ophthalmol. 1991;75(1):53. 1991089 10.1136/bjo.75.1.53 PMC504108

[3] Sternberg P Jr., Machemer R. Subretinal proliferation. Am J Ophthalmol. 1984;98(4):456–62. 6486219 10.1016/0002-9394(84)90131-4

[4] Lewis H, Aaberg TM, Abrams GW, McDonald HR, Williams GA, Mieler WF. Subretinal membranes in proliferative vitreoretinopathy. Ophthalmology. 1989;96(9):1403–14. discussion 14– 5. 2780008 10.1016/s0161-6420(89)32712-6

[5] Shroff D, Saha I, Bhatia G, Dutta R, Gupta C, Shroff CM. Tug of war: a bimanual technique for anterior circumferential proliferative vitreoretinopathy in recurrent retinal detachment. Indian J Ophthalmol. 2020;68(10):2155–8. 32971629 10.4103/ijo.IJO_2179_19 PMC7727933

[6] Charles S. In: SCHACHAT AP, editor. Principles and techniques of vitreoretinal surgery. Ryan’s RETINA: Elsevier Inc.; 2018. pp. 1916–32.

[7] Chang EY, Williams G. Subretinal bands in proliferative vitreoretinopathy. November/December: Retina Today; 2012.

[8] Coffee RE, Jiang L, Rahman SA. Proliferative vitreoretinopathy: advances in surgical management. Int Ophthalmol Clin. 2014;54(2):91–109. 24613887 10.1097/IIO.0000000000000023

[9] Fan W, Shen H, Su N, Yuan S. Transscleral incision for peeling subretinal proliferation tissue in grade C proliferative vitreoretinopathy. Retina. 2023;43(11):2045–50. 35030148 10.1097/IAE.0000000000003391 PMC10589422

[10] Wolff B. Subretinal surgery Ab externo: a novel approach to access the subretinal space without the need for retinotomy. Retina. 2015;35(7):1474–5. 25932551 10.1097/IAE.0000000000000593

[11] Kaga T, Yokoyama S, Kojima T, Mitamura H, Mori T, Matsuda T, et al. Novel endoscope-assisted vitreous surgery combined with atmospheric endoscopic technique and/or subretinal endoscopic technique for rhegmatogenous retinal detachment with grade C proliferative vitreoretinopathy. Retina. 2019;39(6):1066–75. 29528982 10.1097/IAE.0000000000002121

[12] Tang Y, Wu R. Transscleral removal of subretinal strand without vitrectomy: a case report. Case Rep Ophthalmol. 2021;12(3):766–72. 34720975 10.1159/000516850 PMC8525306

[13] Sabti KA, Raizada S. Novel surgical pathway for controlled access to the subretinal space: a case series. Transl Vis Sci Technol. 2022;11(4):11. 35416947 10.1167/tvst.11.4.11 PMC9012894

[14] Hahn P. Anterior Proliferative Vitreoretinopathy With Recurrent Retinal Detachment, Hypotony, and IOL Dislocation. In: Supplement to Retina Today. 2017. Accessed 31 May 2025. https://assets.bmctoday.net/retinatoday/pdfs/0917_supp4.pdf

[15] The classification of. Retinal detachment with proliferative vitreoretinopathy. Ophthalmology. 1983;90(2):121–5. 6856248 10.1016/s0161-6420(83)34588-7

[16] Silpa-Archa S. Sutureless and equipment-free technique for contact lens viewing system during vitreoretinal surgery. Retina. 2023;43(7):1204–6. 32769715 10.1097/IAE.0000000000002933

